# Ginsenoside-Rg1 Protects the Liver against Exhaustive Exercise-Induced Oxidative Stress in Rats

**DOI:** 10.1155/2012/932165

**Published:** 2011-09-20

**Authors:** Mallikarjuna Korivi, Chien-Wen Hou, Chih-Yang Huang, Shin-Da Lee, Ming-Fen Hsu, Szu-Hsien Yu, Chung-Yu Chen, Yung-Yang Liu, Chia-Hua Kuo

**Affiliations:** ^1^Laboratory of Exercise Biochemistry, Department of Sports Sciences, Taipei Physical Education College, Taipei 11153, Taiwan; ^2^Department of Health and Nutrition Biotechnology, Asia University, Taichung 41354, Taiwan; ^3^Graduate Institute of Chinese Medicine, China Medical University, Taichung 40402, Taiwan; ^4^The Chest Department, Taipei Veterans General Hospital and School of Medicine, National Yang-Ming University, Taipei 112, Taiwan; ^5^Department of Physical Therapy, Graduate Institute of Rehabilitation Science, China Medical University, Taichung 40402, Taiwan

## Abstract

Despite regular exercise benefits, acute exhaustive exercise elicits oxidative damage in liver. The present study determined the hepatoprotective properties of ginsenoside-Rg1 against exhaustive exercise-induced oxidative stress in rats. Forty rats were assigned into vehicle and ginsenoside-Rg1 groups (0.1 mg/kg bodyweight). After 10-week treatment, ten rats from each group performed exhaustive swimming. Estimated oxidative damage markers, including thiobarbituric acid reactive substance (TBARS) (67%) and protein carbonyls (56%), were significantly (*P* < 0.01) elevated after exhaustive exercise but alleviated in ginsenoside-Rg1 pretreated rats. Furthermore, exhaustive exercise drastically decreased glutathione (GSH) content (*∼*79%) with concurrent decreased superoxide dismutase (SOD), catalase (CAT), and glutathione peroxidase (GSH-Px) activities. However, these changes were attenuated in Rg1 group. Additionally, increased xanthine oxidase (XO) activity and nitric oxide (NO) levels after exercise were also inhibited by Rg1 pretreatment. For the first time, our findings provide strong evidence that ginsenoside-Rg1 can protect the liver against exhaustive exercise-induced oxidative damage.

## 1. Introduction

 It is well documented that regular exercise has many health beneficial effects including improvement of antioxidant status in liver [1, 2]. However, acute bout of exhaustive exercise can produce a large quantity of reactive oxygen species (ROS) due to increased oxygen consumption [3]. In addition to the mitochondrial respiratory chain, activated xanthine oxidase (XO) is an another source for free radicals/ROS generation [4]. ROS are scavenged by a sophisticated antioxidant defense system, which includes enzymes, superoxide dismutase (SOD), catalase (CAT) and glutathione peroxidase (GSH-Px), and nonenzyme glutathione (GSH) [5]. However, when ROS exceeds the normal physiological coping range during exhaustive exercise, accumulation of ROS and decrease in antioxidant status could be resulted. This scenario increased oxidative stress and leads to modification of lipid and protein structures that consequently compromises the cellular functions in liver [3, 4, 6, 7]. 

**Figure 1:**
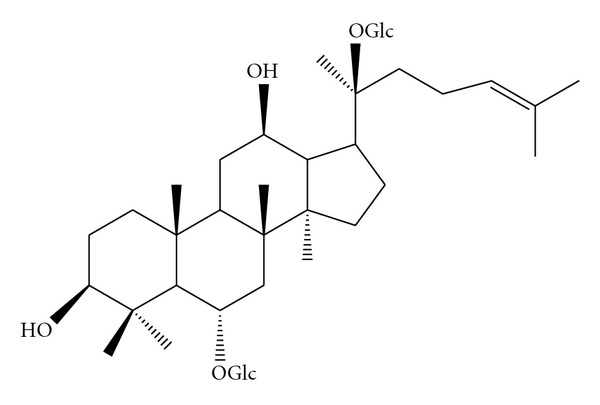
Chemical structure of ginsenoside-Rg1.

From the ancient times, one of the popular herb, *Panax ginseng *and its derivatives has been used as a hepatoprotective remedy in Chinese herbal medicine [8]. However, effect of* Panax ginseng* extracts against free radicals attack has previously reported mixed results [9, 10]. The major biologically active compounds in ginseng are ginsenosides. The ginsenosides are responsible for the pharmacological actions of *Panax ginseng*, which contains a diverse group of steroidal saponins composed of Rb, Rc, Re, Rg, and Rh compounds [8, 11]. Pharmacological actions of these ginsenosides are different, and some of them even showed the opposing effect [9, 10]. Furthermore, ginsenoside profile from the same species could also change due to season, geographical location, and soil [8, 12–14]. Thus, inconsistent ginsenoside profile due to these confounding factors can yield contradictory results when the crude ginseng extract was used as a medicine. To circumvent this ambiguity, use of pure ginsenoside would be the best method to determine the biological action of *Panax ginseng.* Among various ginsenosides, Rg1 (Figure 1) is one of the most active and abundant steroid saponin that shares structural similarity with many steroid hormones [12, 15]. Rg1 has been found as an antioxidant substance [16] and attenuated the oxidative damage in liver of thioacetamide treated rats [17].

In this context, it is currently unknown whether ginsenoside-Rg1 compound is able to regulate the free-radical scavenging system and protect the liver against exercise-induced oxidative damage. Therefore, this study was designed to investigate the protective effects of ginsenoside-Rg1 against exhaustive exercise-induced disturbed antioxidant status and oxidative stress in the liver of rats.

## 2. Materials and Methods

### 2.1. Plant Extract and Chemicals

All the chemicals used in this study were obtained from Sigma Chemicals, USA. Ginsenodie-Rg1 (molecular weight 801.01, Figure 1) was obtained from the NuLiv Science USA, Inc, Walnut, Calif, USA.

### 2.2. Exercise Protocol

In our study, 4-month aged rats were subjected to exhaustive swimming exercise in a water pool. The water temperature was maintained between 33°C and 34°C, and keen observation was taken to perform the exhaustive exercise. During exercise, each rat was loaded with 3% additional weight according to its body weight in order to get the exhaustive performance. No significant difference in swimming hours was noticed between groups. Prior to exercise performance, rats were allowed to swim for 10–15 min for 3 days to familiarize the swimming environment.

### 2.3. Animal Care and Maintenance

Healthy male Sprague Dawley (SD) rats (*n* = 40) weighing 410 ± 10 g were maintained under a temperature-controlled room (22 ± 2°C) with 55% humidity and photoperiod of 12-h light and 12-h dark cycle. All the rats were housed in the clean polypropylene cages under hygienic conditions and allowed free access to standard laboratory chow (PMI Nutrition International, Brentwood, Mo, USA) and water *ad libitum*. This study was approved by the Animal Care and Use Committee of Taipei Physical Education College, and conformed to the Guidelines for the “Use of Research Animals” published by the Council of Agriculture, Executive Yuan, Taiwan.

### 2.4. Experimental Design and Treatment

Weight matched rats were assigned into two groups, twenty in each group, and treated as follows.

**Vehicle**

In this group, all rats (*n* = 20) received 0.9% saline via orogastic tube for 10-week in order to equivalent handling with treated animals.

**Ginsenoside-Rg1**

This group of rats (*n* = 20) were treated with the purified ginseng extract, ginsenoside-Rg1 at the dose of 0.1 mg/kg body weight per day via orogastric tube for a period of 10 weeks. Prior to administration, ginsenoside-Rg1 compound was dissolved in saline (0.9%) and prepared the dose equivalent to 0.1 mg/kg body weight.

After completion of the last day of treatment, ten rats from each group performed the exhaustive swimming exercise as described in the “exercise protocol”. In this study, we considered resting rats as nonexercise and exhaustive exercised rats as exercised/postexercise rats in both groups. Immediately after exhaustive performance, all the rats were anaesthetized with chloral hydrate (400 mg/kg body weight, intraperitoneal), and liver tissue was quickly excised. The collected tissues were washed thoroughly with saline to remove the excessive blood from liver then, immediately frozen in to liquid nitrogen and stored at −80°C until further biochemical evaluations.

### 2.5. Biochemical Evaluations

#### 2.5.1. Determination of Lipid Peroxidation and Protein Oxidation

Tissue lipid peroxidation was monitored by screening the thiobarbituric acid reactive substances (TBARS) as described in the Cayman's TBARS assay kit. Under high temperature (90°C–100°C), malondialdehyde (MDA) combined with thiobarbituric acid (TBA) and formed MDA-TBA adducts that were measured at 540 nm in a spectrophotometer. Protein oxidation in the liver sample was determined by measuring the protein carbonyl residues by using the DNPH (2,4-dinitrophenylhydrazine). According to the Cayman's protein carbonyl assay kit protocol, the amount of protein-hydrozone product was quantified spectrophotometrically at wavelength of 360 nm (Tecan Genios, A-5082, Austria).

#### 2.5.2. Estimation of Antioxidant Enzyme Activities

Superoxide dismutase (SOD) activity in the liver samples was estimated by using the xanthine oxidase, and the absorbance was read at 450 nm using ELISA plate reader. One unit of SOD is defined as the amount of enzyme needed to exhibit 50% dismutation of the superoxide radical. Hepatic catalase (CAT) activity was determined by adding the hydrogen peroxide (H_2_O_2_) to the samples and the absorbance was read at 540 nm using 96-well plate reader (Tecan Genios, A-5082, Austria). 

Both glutathione peroxidase (GSH-Px) and glutathione reductase (GR) enzyme activities were measured in accordance with the protocol supplied by the manufacturer. The decreased in the absorbance of the oxidation of NADPH was measured at 340 nm once every minute to obtain at least 5 time points using a plate reader (Tecan Genios, A-5082, Austria). Enzyme activities were calculated per mg protein, and protein concentrations in the liver homogenates were estimated by BioRad protein assay reagent (Richmond, Calif, USA) as described in the manufacturer instructions. All these antioxidant enzymes were assayed by the kits of Cayman Chemical Company, USA.

#### 2.5.3. Estimation of Reduced Glutathione Levels

The accurate reduced glutathione (GSH) concentration in the liver sample was estimated by using the DTNB (5,5′-dithiobis-2-nitrobenzoic acid) according the protocol provided by the commercial kit's (Cayman). The absorbance of the sample was read at 405 nm using the 96-well plate at 5 min interval for 30 min in a spectrophotometer.

#### 2.5.4. Xanthine Oxidase Activity Assay

Liver xanthine oxidase (XO) activity was assayed based on the multistep enzymatic reaction with the corresponding substrate using commercial kit (Cayman). The fluorescence of the sample was detected at an excitation wavelength of 535 nm and an emission wavelength of 590 nm in a fluorescence spectrophotometer (Tecan Genios, A-5082, Austria). The enzyme activity was calculated per mg protein.

#### 2.5.5. Measurement of Nitric Oxide (Nitrate/Nitrite) Concentration

Accurate nitric oxide (NO) concentrations through its stable metabolites (nitrate and nitrite) were determined spectrophotometrically by using the commercial kits (Cayman). In this process, first nitrate was converted in to nitrite-by-nitrate reductase. In the second step, nitrite was converted to deep-purple azo product by adding the Griess reagent, and the absorbance was read at 540 nm using ELISA plate reader (Tecan Genios, A-5082, Austria).

### 2.6. Statistical Analysis

The data obtained from this study was analyzed by using the SPSS software, and results were expressed as mean ± SE for ten replicates. One-way analysis of variance (ANOVA) was carried out to compare the significance followed by Tukey's multiple comparisons test, and *P* value less than 0.05 was considered statistically significant. 

## 3. Results

### 3.1. Role of Ginsenoside-Rg1 against Lipid Peroxidation and Protein Oxidation

**Figure 2:**
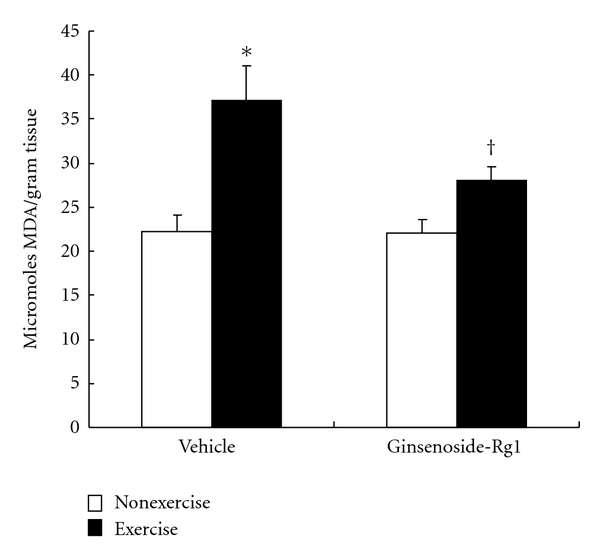
Effect of ginsenoside-Rg1 pretreatment on TBARS levels after exhaustive exercise in liver of rats. Values are significant compared to vehicle nonexercise (*P < 0.001) and vehicle exercise groups (^†^ *P* < 0.05).

Lipid peroxidation widely considered as an oxidative stress marker, caused by excessive amount of free radicals attacking on membrane components of the cell. In this study, lipid peroxidation marker estimated as TBARS levels were significantly (*P *< 0.001) elevated (67%) in vehicle group after exhaustive exercise performance (Figure 2). An important finding of the present study is that exercise-induced elevated TBARS levels were significantly controlled in ginsenoside-Rg1 pretreated rats. Nevertheless, no significant difference in TABARS levels were observed between vehicle and ginsenoside-Rg1 pretreated groups at resting condition. 

**Figure 3:**
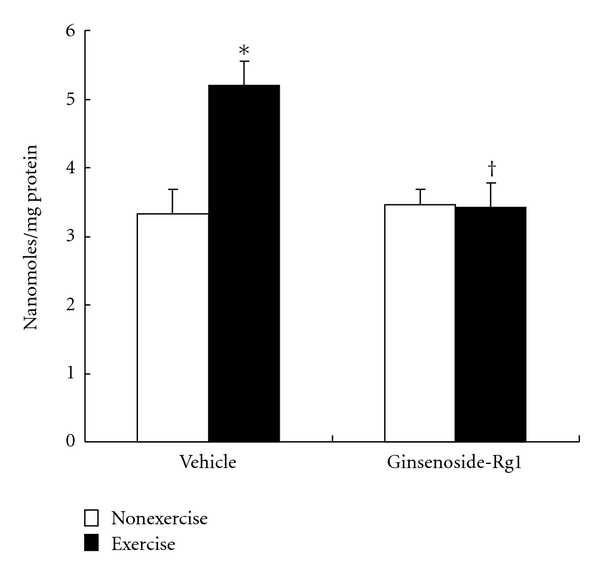
Effect of ginsenoside-Rg1 pretreatment on protein carbonyl residues after exhaustive exercise in liver of rats. Values are significant compared to vehicle nonexercise (*P < 0.001) and vehicle exercise groups (^†^ *P *< 0.001).

Protein oxidation estimated in terms of protein carbonyl residues were significantly (*P* < 0.001) elevated after exhaustive exercise in vehicle group (Figure 3). It is noteworthy that exhaustive exercise-induced elevation in protein carbonyl level (56%) was not found in ginsenoside-Rg1 pretreated rats. Furthermore, the values in Rg1 treated rats after exercise was significantly (*P* < 0.01) lower than exercised rats in vehicle group.

### 3.2. Effect of Ginsenoside-Rg1 on Antioxidant Enzyme Activities

**Figure 4:**
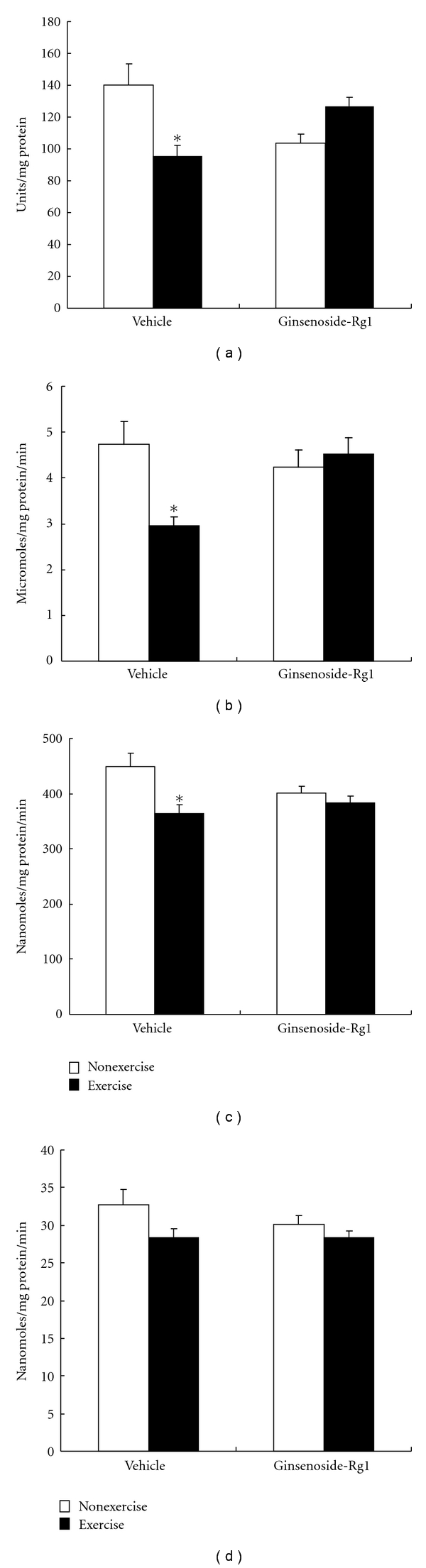
Effect of ginsenoside-Rg1 pretreatment on various antioxidant enzyme activities including SOD (a), CAT (b), GSH-Px (c), and GR (d) after exhaustive exercise in liver of rats. Values are significant compared to vehicle nonexercise group (*P < 0.01).

In the present study, exhaustive exercise significantly (*P* < 0.01) decreased liver SOD activity compared to nonexercised rats in vehicle group (Figure 4(a)). However, this decrease was not observed in ginsenoside-Rg1 pretreated rats after exhaustive exercise. Furthermore, instead of decrease, SOD activity showed increased trend (~23%) in ginsenoside-Rg1 pretreated rats after exhaustive exercise. 

Similar to SOD, liver CAT activity was also significantly (*P* < 0.01) decreased (37%) in vehicle group after exhaustive exercise (Figure 4(b)). Although administration of ginsenoside-Rg1 alone had no effect on CAT activity, here, we found that exhaustive exercise-induced decrease was attenuated by ginsenoside-Rg1 pretreatment. CAT activity was almost reached to normal levels in ginsenoside-Rg1 pretreated exercised rats. 

GSH-Px and GR are the two enzymes responsible for maintaining the stable intracellular GSH levels. Alterations in these enzyme activities were represented in Figures 4(c) and 4(d). Exhaustive exercise significantly (*P *< 0.01) decreased GSH-Px activity in vehicle group, while no significant change in GR activity. Nevertheless, pretreatment of Rg1 maintains the stable GSH-Px activity even after exhaustive exercise.

### 3.3. Effect of Ginsenoside-Rg1 on GSH Levels

**Figure 5:**
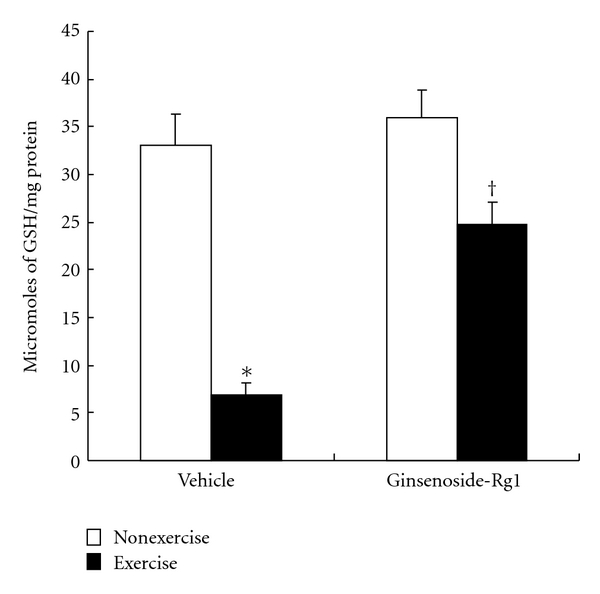
Effect of ginsenoside-Rg1 pretreatment on glutathione (GSH) concentrations after exhaustive exercise in liver of rats. Values are significant compared to vehicle nonexercise (*P < 0.001) and vehicle exercise groups (^†^ *P *< 0.01).

Exhaustive exercise drastically (*P* < 0.001) decreased the intracellular GSH concentrations in vehicle group. The drop in GSH content was **~**79% in exhaustive exercise rats. Interestingly, we found no significant decrease in ginsenoside-Rg1 pretreated rats after exhaustive exercise performance. In addition, GSH levels were significantly higher in Rg1 pretreated exercised rats compared to vehicle group exercised rats (Figure 5).

### 3.4. Impact of Ginsenoside-Rg1 on Xanthine Oxidase Activity and Nitric Oxide Levels

**Figure 6:**
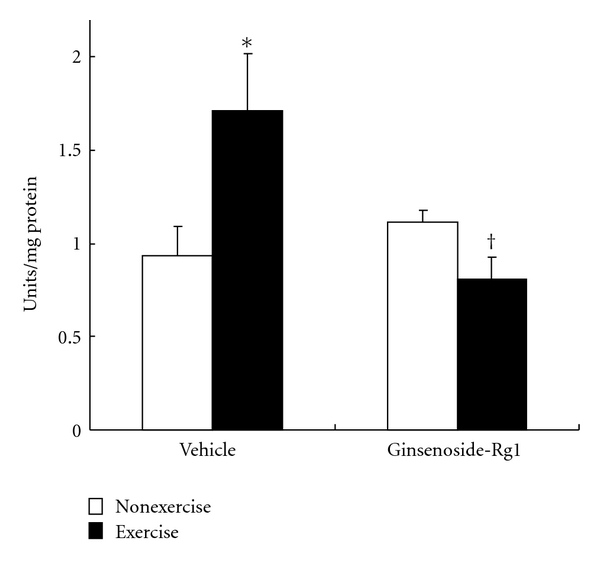
Effect of ginsenoside-Rg1 pretreatment on xanthine oxidase (XO) activity after exhaustive exercise in liver of rats. Values are significant compared to vehicle nonexercise (*P < 0.001) and vehicle exercise groups (^†^ *P *< 0.01).

XO activity was measured in the liver to address whether XO plays a prominent role in free-radical production during exhaustive exercise. Here, we found a profound (*P *< 0.001) increase in XO activity (~83%) after exhaustive exercise in vehicle group. Important finding of the study is that elevated XO activity was abolished in ginsenoside-Rg1-treated exercised rats. The decreased XO was about 2-fold in ginsenoside-Rg1 pretreated rats after exhaustive exercise compared to vehicle group exercised rats (Figure 6). 

**Figure 7:**
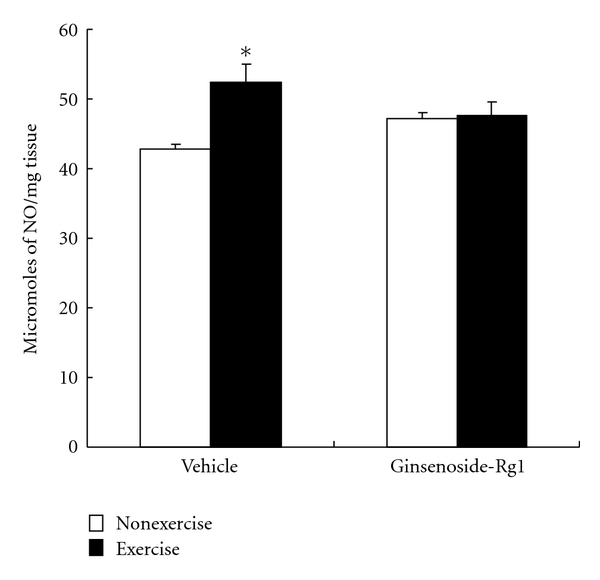
Effect of ginsenoside-Rg1 pretreatment on nitric oxide (NO, nitrate/nitrite) concentrations after exhaustive exercise in liver of rats. Values are significant compared to vehicle nonexercise group (*P < 0.05).

Estimated nitric oxide (NO) levels as nitrate/nitrite concentrations were significantly (*P* < 0.05) increased in the liver of exercised rats (Figure 7). Increased NO levels further confirms the exhaustive exercise-induced tissue damage in liver. Rats received ginsenoside-Rg1 substance for 10-week showed no significant change in NO levels after exercise, which represents exercise-induced elevated NO levels were inhibited by Rg1 pretreatment in liver.

## 4. Discussion

An acute bout of exhaustive exercise not only increases free-radical production [3] but also impairs free-radical scavenging system [7, 18], which exacerbates liver capability to buffer high amount of radicals. In this study, we found that prolonged use of ginsenoside-Rg1 significantly attenuated the exhaustive exercise-induced adverse effects in liver antioxidant status. This was evidenced by substantially less amount of TBARS and protein carbonyls in Rg1 pretreated exercised rats, compared to vehicle group. The suppressed oxidative damage is well explained by (1) restored GSH levels, (2) attenuated antioxidant enzyme activities including SOD, CAT, and GSH-Px, and (3) inhibited XO activity and NO levels in Rg1 treated exercised rats. These findings provide evidence that Rg1, the major ginsenoside component of *Panax ginseng*, is able to reinforce the hepatic free-radical buffering system against exhaustive exercise challenge. 

Exhaustive exercise-induced elevated TBARS indicates increased membrane lipid peroxidation in liver. Davies et al. [3] showed elevated lipid peroxidation by increased free radicals in rat liver after exhaustive exercise. Increased membrane peroxidation ultimately affect cell structure and functions and cause tissue damage [7, 19, 20]. However, in this study, no such increased peroxidation was noticed in ginsenoside-Rg1 supplemented exercised rats. Previously, Geng et al. [17] reported attenuated hepatic TBARS by Rg1 in thioacetamide treated rats. Voces and colleagues [21] demonstrated reduced hepatic lipid peroxidation by *Panax ginseng* extracts in exhaustive exercised rats. Ginseng saponins has been shown to decrease phospholipase A_2_ (PLA_2_) activity [22], which is responsible for lipid peroxidation [23]. On the other hand, improved antioxidant status by Rg1 may capture the excessive free radicals and aid to terminate the chain reaction of lipid peroxidation, thus protect the liver cells from membrane damage. 

Exercise-induced oxidative damage to proteins in liver was accompanied by elevated protein carbonyls (PC). It is well documented that excessive superoxide anion (O_2_^•−^) and hydroxyl radicals (^−^OH) resulted from exhaustive exercise react with proteins, enhance the oxidative damage [3, 18]. Another interesting finding from our study is that elevated protein carbonyls were significantly reversed by ginsenoside-Rg1 pretreatment. Although ginseng extracts showed reduced protein carbonyls against eccentric exercise in rat muscle [24], for the first time, we demonstrated decreased protein oxidation by Rg1 in liver against exhaustive exercise. The underlying mechanism behind this protection is still unclear. However, it is possibly due to inhibition of excessive ROS formation and/or intracellular Ca^+^ levels by Rg1, since Rg1 has been proven as a potent inhibitor of ROS and intracellular Ca^+2^ levels [17, 25]. In addition, ginseng extracts also reported as free-radical scavenger [8, 9], which reveals excessive ROS-induced protein oxidation might be ameliorated by preadministration of ginsenoside-Rg1 in liver. 

Hepatic superoxide dismutase (SOD) plays a key role in removal of O_2_^•−^ to H_2_O_2_, which was significantly decreased after exhaustive exercise. It is well demonstrated that exhaustive exercise trigger the O_2_^•−^ production either by increased oxygen consumption or by activated XO activity [3, 4]. To counter excessive O_2_^•−^ induced hepatic cell death [26], SOD utilization may increase and combat against toxic O_2_^•−^ that apparently decrease the net activity. However, ginsenoside-Rg1 prevented this loss against exercise. As an antioxidant, Rg1 has been shown to inhibit the activation and formation of free radical in hepatic stellate cells (HBCs) [17]. We also found inhibited XO, a free-radical source, after exercise in Rg1 pretreated rats. Due to its unique antioxidant property, we speculate that ginsenoside-Rg1 potentially scavenge O_2_^•−^ radicals, therefore, maintain the net SOD activity and protect liver cells against O_2_^•−^ during exhaustive exercise. Catalase (CAT), responsible enzyme for decomposition of H_2_O_2_ was significantly decreased after exhaustive exercise in liver, thereby results in the accumulation of H_2_O_2_. CAT activity was expected to decrease to buffer the excessive H_2_O_2_. In line with our findings, Sureda et al. [27] found decreased CAT in professional cyclists participated in a mountain stage. However, ginsenoside-Rg1 pretreatment takeover the exercise impact and restored CAT activity, which suggests accumulated H_2_O_2_ may effectively eradicated by Rg1. Previous study indicated that ginsenoside protopanaxatriol, contains Rg1, may affect the genomic expression of CAT against H_2_O_2_-induced injury in endothelial cells [11]. Furthermore, ginsenoside-Rg1 has been showed to suppress intracellular Ca^+^ levels besides increased CAT activity in cardiomyocyte against hypoxia/reoxygenation oxidative injury [25]. In our findings, augmented CAT activity along with SOD by Rg1 may attenuate the exercise-induced oxidative injury in liver. 

Decreased GSH-Px activity after exhaustive swimming in vehicle group made liver susceptible to oxidative stress. Increased ROS production and drop in GSH content in response to exhaustive exercise [28], which was also reported in this study, may be associated with decreased GSH-Px activity. Here, it is interesting to note that Rg1 is capable to handle the fluctuations in intracellular redox status by maintaining the stable GSH-Px activity. Supporting evidences demonstrated that ginsenoside protopanaxatriol could activate GSH-Px activity against H_2_O_2_ exposure in endothelial cells [11]. Ginseng extract-G115 improved liver GSH-Px activity in rats subjected to exhaustive treadmill exercise [21]. Additionally, Rg1 improved GSH content along with primary antioxidant enzymes and suppressed ROS formation [11, 17, 25] or both are possible explanations for the restored GSH-Px activity in liver. In contrast glutathione reductase (GR) activity was not significantly altered either by exhaustive exercise or by ginsenoside-Rg1 supplementation. Previous studies also reported no exhaustive exercise effect on liver GR activity in rats [7, 19]. It has been argued that different ginsenosides have different effect on GR activity. Ng and Yang [10] found no significant change in GR activity with ginsenoside-Rg3 exposure in C6 glioma cells. While, Kwok et al. [11] observed increased GR activity with Rg1 treatment in endothelial cells against H_2_O_2_ exposure. 

As expected, liver GSH levels were drastically decreased after exhaustive exercise, since liver cells utilizes most of GSH to eliminate the high amount of ROS. Acute exercise-induced decreased cystine, a rate-limiting precursor for GSH synthesis may be responsible for the depleted GSH levels [6]. Decreased GSH concentrations in liver is an index of mild-to-severe oxidative stress [6, 18, 21, 28], which was also evidenced by increased TBARS and protein carbonyls in this study. The most important finding from our study is that decreased GSH content was not observed in Rg1 pretreated exercised rats, which presumably reflects improved antioxidant status in liver. Evidence showed that ginsenoside-Rg1 can improve GSH-cycle enzymes and protect the cells from H_2_O_2_-induced cell death [29]. Furthermore, Kwok et al. [11] found restored GSH levels in H_2_O_2_-treated endothelial cells by protopanaxatriol pretreatment. Taken together, our findings demonstrate that Rg1 pretreatment can counter the exercise-induced oxidative stress, and protect the liver cells through stabilizing GSH levels. 

Similar to earlier reports, elevated XO activity in liver after exercise is considered as a main intracellular source for O_2_^•−^ production [19, 30], since we found decreased SOD activity in this study. During exercise, resynthesis of ATP from ADP may occur through adenylate kinase reaction, simultaneously formed AMP degraded to hypoxanthine and released into blood stream. Hypoxanthine produced by skeletal muscle is taken up by the liver and further oxidized to uric acid, which indicates activated XO activity [4, 31]. Nevertheless, elevated XO was completely inhibited in Rg1 treated rats. Similarly, administration of ginsenoside-Rg1 reversed the activated XO and impaired SOD activities in mice against glutamate-toxicity [32]. From these evidences, we speculate that prolonged use of Rg1 may suppress intracellular Ca^+^ levels that results suppressed XO activity in exercised rats. 

Proper maintenance of intracellular NO is crucial to protect the liver against NO-mediated oxidative stress. Besides its beneficial effects, high levels of NO reacts with O_2_^•−^ and form peroxynitrite (ONOO−), a potent oxidant, capable to attack lipids and proteins and further contributes oxidative damage [33, 34]. This was evidenced by increased TBARS and protein carbonyls in exercised rat liver. Exercise-induced increased NO levels were well documented by previous reports [24, 35]. Inhibited NO levels by Rg1 indicates its capabilities to attenuate the oxidative damage caused by high levels of NO in liver. A study demonstrated that pretreatment of Rg1 attenuated the dopamine-induced elevation of ROS or NO generation, eventually protect the mitochondria from ROS-mediated injury [36]. Our findings clearly indicate that Rg1 offers protection against oxidative stress and maintain the stable antioxidant status.

## 5. Conclusions

The present study provides evidence that pretreatment of ginsenoside-Rg1 attenuated the exhaustive exercise-induced oxidative stress in liver of rats. To our knowledge, for the first time, we demonstrated that oral administration of ginsenoside-Rg1 profoundly decreased XO and NO levels with a consistent suppressed TBARS and PC levels, and restored antioxidant status against exhaustive exercise. Our results revealed that Rg1 is capable to buffer the excessive free radicals and attenuate the oxidative damage in liver. Taken together, these results suggest that Rg1 can be used as an antioxidant supplement for competing athletes, who participates in exhaustive endurance events. However, related human studies are encouraged to prescribe the ginsenoside-Rg1 as a nutraceutical supplement to athletes.
